# Effects of Forced Alcohol Intake Associated with Chronic Stress on the Severity of Periodontitis: An Animal Model Study

**DOI:** 10.1155/2012/465698

**Published:** 2012-10-31

**Authors:** Alessandra Nogueira Porto, Alex Semenoff Segundo, Tereza Aparecida Delle Vedove Semenoff, Fabio Miranda Pedro, Álvaro Henrique Borges, José Roberto Cortelli, Fernando de Oliveira Costa, Sheila Cavalca Cortelli

**Affiliations:** ^1^Nucleus of Periodontal Research, University of Taubaté, 12020-340 Taubaté, SP, Brazil; ^2^Dental School, University of Cuiabá, 78020-590 Cuiabá, MT, Brazil; ^3^University of Cuiabá, 78020-590 Cuiabá, MT, Brazil; ^4^Department of Periodontology, Federal University of Minas Gerais, 31270-901 Belo Herizonte, MG, Brazil

## Abstract

This study histometrically evaluated the effect of forced alcohol intake by stressed animals on the severity of ligature-induced periodontitis in rats. Thirty-two rats were randomly divided in four groups: group GAL—alcohol and ligature; group GASL—alcohol, chronic physical stress, and ligature; GNC—negative control; GPC—positive control. GAL and GASL received 20% ethanol *ad libitum*, and GNC received water *ad libitum* for 60 days. After 24 hours of exposition to alcohol intake—by GAL and GASL—immobilization was applied as a chronic stressor in the GASL group for a two-month period, six times a week, in random hours. The means of the respective groups were statistically compared (Analysis of Variance and Tukey tests, *P* < 0.05). The most severe periodontal breakdown was observed in nonstressed animals which drank alcohol (GAL), followed by stressed animals exposed to alcohol (GASL). GASL did not differ from the positive control group (GPC). The negative control group showed the lowest values of periodontal breakdown (*P* < 0.05). *Conclusions*. Non-stressed alcohol consumer animals showed the most severe pattern of periodontal breakdown. Although stressed animals which were forced to drink alcohol showed poorer periodontal status than the negative controls, their results were similar to those of positive controls.

## 1. Introduction

Periodontal disease is a multifactorial infectious disease triggered by the development of dental biofilm, which can harm periodontal tissues. There are several factors which influence periodontitis: personal and social characteristics, behavioral, systemic, genetic and dental factors, and the microbial composition of the dental biofilm [1, 2].

Stress may be defined as the combination of physical and mental responses caused by certain external stimuli (stressors) which allow an individual (human or animal) to overcome certain environmental demands, and the physical and mental weariness caused by the stress process itself [3]. Stress has been related not only to a higher occurrence of periodontal disease but also to its severity [4]. Other social and behavioral factors that could be related to periodontal status are tobacco use, social economic status, nutritional status, psychological aspects, and abusive alcohol intake [1]. Among these factors, alcohol has become relevant because it is one of the few socially accepted psychotropic drugs. This social acceptance has contributed to a rise in alcoholism, which has become a serious public health problem [5]. According to Silva Furtado Amaral et al. [6] the World Health Organization has reported that the rates of death and functional limitations associated with abusive alcohol intake have surpassed those associated with tobacco smoking. Alcoholism per se imposes a state of stress because, besides its physiological demand, it acts as a chemical aggressor to the body. All the alcoholic drinks consumed by an individual do not remain stored in the body and, before being eliminated, it is first metabolized in the liver, which results in high levels of reactive oxygen species [7, 8].

Epidemiological studies have related periodontal disease to the consumption of alcoholic drinks. Several cross-sectional studies have verified an association between the abusive consumption of alcohol and poor periodontal health [9–11]. Abusive consumption of alcohol has also been associated with the severity of periodontitis [2]. However, many questions on this subject are still not answered and laboratorial research could be helpful to clarify these doubts. Animal model studies may more specifically help elucidate questions related to alcohol intake, stress, and periodontal disease [12, 13].

As a lifestyle factor, alcohol intake could vary under stressful situations. As isolate factors, drinking, and stress seem to contribute to poorer periodontal status. In the present study it was hypothesized that alcohol consumption by stressed animals could be accompanied by an increase in the severity of periodontitis. Therefore, aiming to confirm or reject this hypothesis we evaluated whether forced alcohol intake by stressed animals affects histometrical parameters of periodontal breakdown.

## 2. Materials and Methods

Thirty-two animals of the *Rattus norvegicus* species *Lewis* breed (two-month old), weighing average 250 g, were selected for the present study. All the selected animals went through an environmental adaptation period of four weeks. The animals were divided in groups of four and each group was kept in a cage (polyethylene 16 × 40 × 30) [14, 15]. They all received standard rat feed and water or alcohol (ethanol 20%) *ad libitum*. They were kept in light/dark cycles of 12 hours; the temperature was controlled at 24°C and humidity at >50%. The experiment took place at the University Center of Várzea Grande—UNIVAG and it was approved and registered by the ethics in animal research committee (CEP/UNIC-2009 no. 307-321) of the Institution.

### 2.1. Experimental Groups

Initially, a research assistant randomly divided the animals in four experimental groups:

1. group GAL: alcohol + ligature (*n* = 8);
2. group GASL: alcohol + stress + ligature (*n* = 8);
3. group GPC: stress + ligature, positive control (*n* = 8);
4. group GCN: negative control (*n* = 8).

After the division, the animals in the GAL and GASL groups were offered alcohol (ethanol 20%) *ad libitum* for 60 days [11, 16] while negative control (GCN) received water *ad libitum* for the same period of time.

### 2.2. Experimental Periodontal Disease

Because alcohol intake is very limited on the first day, ligatures were inserted only in the second experimental day (GAL, GASL, and GPC). The animals received general anesthesia through the intramuscular administration of 0.05 mL xylazine hydrochloride (Rompun, Bayer. Animal Health, São Paulo, SP. Brazil) and 0.1 mL ketamine (Dopalen, Agribrands. Animal Health, Paulínia, SP, Brazil) per 100 grams of body weight.

After anesthesia, a sterile silk suture number 4 (Ethicon, Johnson and Johnson, São Paulo, SP, Brazil) was placed in the gingival crevice of the second right upper molar [17].

The animals in the positive control group were kept in the same environment in the first experimental day, drinking water, but without suffering any kind of intervention. The animals in the negative control group (GNC) did not receive any type of intervention but were kept in their cages in the same environment during all the study.

### 2.3. Stress Induction

The model of physical stress induction chosen for this study was immobilization done during 60 days, six times a week, in different times of the day as previously described [18, 19]. At room temperature, the animals in the GASL group were placed in polyvinyl chloride tubes compatible to their body sizes. The tubes were closed on both ends with metallic wire, which enabled the animals to breathe while they were immobilized for four consecutive hours.

### 2.4. Histological Examination

After sixty days all the animals were euthanized. Right maxilla was removed and fixed in formaldehyde 10% for 48 hours. After that process, the jaw segment was decalcified in EDTA for approximately five weeks (EDTA was renewed six times); following dehydration in graded alcohol, the samples were embedded in paraffin. The tissue blocks included in paraffin allowed 6 *μ*m histological slices that followed the long axis of the teeth in the mesio-distal plane and were stained with hematoxylin and eosin. 

For the histometric analysis, ten serial sections containing the 1st and 2nd upper molars and the following structures (a) dental pulp, (b) the mesial cementoenamel junction of the 2nd molar, (c) proximal bone crest, and (d) connective attachment had to be observed. Histometry was performed in the mesial aspect from first molars, by the same blinded examiner, through the capturing of images in the microscope and measured in millimeters (software ImageLab 2000-Diracon Bio Informática Ltda., SP, Brazil).

### 2.5. Statistical Analysis

The intraexaminer reproducibility was calculated before and during the histometry. In the calibration previous to the study, the error was calculated by double measurements of 10% of the specimens with a one-week interval. During the study, after every 10 examined specimens, one was reexamined. Paired *t*-test statistics were run and no differences were observed in the mean values for comparison (*P* > 0.5). Additionally, Pearson's correlation coefficient was obtained between the 2 measurements in each time point and revealed a very high correlation (0.98 and 0.99; *P* < 0.01). 

 Two histometrical parameters were considered as periodontal status indicators. The distance between the cementoenamel junction and the bottom of the junctional epithelium (CEJ-JE) in the mesial site of the first molars was defined as the loss of histological attachment. In addition, the distance between the cementoenamel junction and the alveolar bone crest was also analyzed and it was considered as the indicator for bone loss (CEJ-BC). The mean values were calculated for each of the groups for further statistical comparisons. Analysis of Variance (ANOVA) and Tukey (*P* < 0.05) tests were applied.

## 3. Results

**Figure 1:**
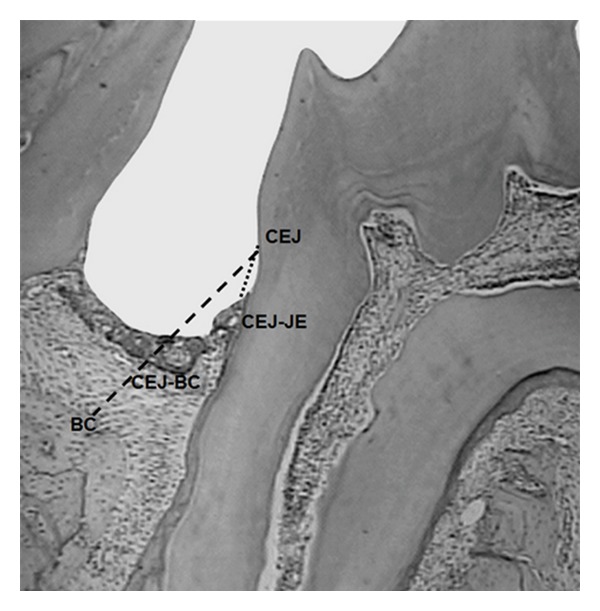
Distance between the CEJ in the mesial side of the second molar and the most apical portion of the junctional epithelium (CRJ-JE) and the distance between the CEJ and the alveolar bone crest (CEJ-BC). 4x magnificence. Image captured from the alcohol associated with stress group (GASL).

**Figure 2:**
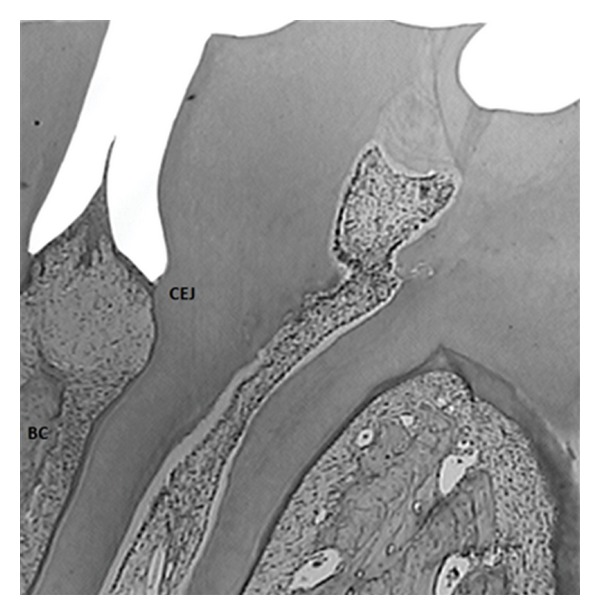
Image captured from the same animal illustrated in Figure 1 (alcohol associated with stress group–GASL) before periodontitis induction.

There were no statistically significant differences among the body weights of the groups at any of the evaluation periods (ANOVA, *P* < 0.05). Loss of histological attachment (CEJ-JE) and bone loss (CEJ-BC) (*P* < 0.05) were statistically different in the animals exposed to alcohol (GAL) and the ones exposed to alcohol and stress (GASL). The most severe periodontal breakdown was observed among the nonstressed animals exposed to alcohol, followed by the group of stressed animals exposed to alcohol. This last group (GASL) did not differ from the positive control group (GPC). Figures 1 and 2 illustrate results from GASL group at two different experimental times. Finally, the negative control group showed the lowest values of CEJ-JE and CEJ-BC when compared to the animals in the other groups (Table 1).

## 4. Discussion

The severity and rate of the progression of periodontitis are influenced by a great variety of determinants and risk factors. Oral hygiene, tobacco smoking, diabetes, age, and the presence of specific microorganisms can be considered as risk factors, since individuals under these conditions seem to be more likely to develop periodontitis [20]. Consistent data in the literature suggests that they represent serious effects over periodontal diseases [21]. However, the combination of these factors is not enough to explain the difference in the progression rate and severity of the disease. Other systemic conditions such as stress [22], osteoporosis [23], obesity [24], and abusive alcohol drinking [6] have been considered as partially responsible for this variability as well.

The effects of alcohol to the body and the functional imbalances associated with its consume have been constantly investigated [25]. Such interest is justified by the wide range of injuries and toxic effects that alcohol drinking causes the organs and tissues, which can reflect in different systemic diseases [26]. Besides, The World Health Organization estimates that two billion people around the world are alcohol consumers, and that 76 million show some kind of systemic adverse event related to that. In Brazil, it is estimated that 68% of the population consumes alcohol and that 11.2% are alcoholics. 

De Souza et al. [27] demonstrated that alcohol consumption directly framed the progression of bone loss in an induced periodontitis model. On the other hand, instead of a direct action of alcohol over the periodontal tissues, these effects are based on poor oral hygiene, which could better explain a higher occurrence and severity of periodontal disease in people who drink alcohol [25]. In this context, animal studies are important as they may help understand the biological events once they allow the elimination of certain bias by the control of some variables. Therefore, animal studies have observed that alcohol affected the periodontal tissues through different mechanisms [28]. Reduced host responses, due to deficiency and functional changes in neutrophils, as well as changes in the protein metabolism and in the healing of tissues have been previously reported [29]. In the present study alcohol drinking negatively impacted the progression of periodontitis. Similarly De Souza et al. [27] also observed a direct effect of alcohol drinking in the bone loss progression in a ligature-induced periodontitis model.

One common question is in which level the alcohol as a water substitute under experimental conditions reflects human alcohol drinking. The answer to that is not simple, especially considering that the metabolism of rats is faster than that of humans. According to The World Health Organization mild drinkers are men who drink 21 alcohol units (1 unit = 10 grams) per week. For women, this number is 14 units per week. For example, a glass of red wine (90 mL) has 12% or 1.7 unit of alcohol. A beer has 5% (1.1 unit) while distilled drinks such as vodka and whisky have 40% or 2.0 alcohol units [5]. Supporting dose dependence, De Souza et al. [30] reported a significantly higher alveolar bone loss in rats receiving 20% ethanol in comparison to rats receiving 10% ethanol or water. Tezal et al. [11] carried an interesting cross-sectional study to evaluate the effect of the consumption of different alcoholic drinks (wine, beer, and liquor) on the severity of periodontal disease. They observed that the effects over the periodontal tissues did not change depending on the kind of drink taken. These same authors also verified contrary activity of alcohol over some periodontal pathogens such as *Actinobacillus actinomycetemcomitans* and *Porphyromonas gingivalis. *Clinical results suggested that alcohol seems to affect more severely the gums, followed by the periodontal ligament and the alveolar bone and that the effect of alcohol on periodontal disease may depend on the dose, frequency, and time of drinking [29].

Shimazaki et al. [9] also demonstrated that individuals that consumed more than 15 grams of alcohol a day had a significant increase in the progression rate of periodontal disease and a higher inflammatory infiltrate, besides a higher number of periodontal pockets when compared to those who did not consume alcohol.

Although, dental biofilm acts besides some recognized risk factors such as tobacco use, as previously mentioned, more recently, researchers are considering whether and in what degree lifestyle could affect the periodontium. In this context, stress seems to affect not only periodontal status but also personal lifestyle. Individuals exposed to stressful situations tended to smoke a higher number of cigarettes [31] and become alcohol consumers [32]. Also, a decrease in oral hygiene was observed in a group of stressed individuals [10]. Furthermore, direct tissue damage could be attributed to the stress process [4]. Due to the frequent combined occurrence of the studied factors, this study evaluated the effect of alcohol intake by stressed animals. The fact that the stressed animals exposed to alcohol showed better periodontal conditions than the nonstressed animals was only a partially unexpected result of this study. Yaroslavsky and Tejani-Butt [33] evaluated the relation between stress and alcohol consumption and observed that rats exposed to chronic stress consumed a greater amount of alcohol. In addition, changes in central dopamine type-2 receptor sites were found indicating an altered dopamine neurotransmission following stress and alcohol exposure. Since stressed animals consumed more alcohol, the authors concluded that it is possible that the consumption of alcohol reverses these alterations related to dopamine, suggesting a self-medicating phenotype. Generally, alcohol intake tends to be confined to the weekends. Jiménez-Ortega et al. [34] compared the immunological effect of the discontinuous feeding of a liquid diet containing a moderate amount of ethanol to that of continuous ethanol administration or a control diet. The discontinuous alcohol group received the ethanol diet for 3 days and the control liquid diet for the remaining 4 days of each week (for a total of 4 weeks). The authors concluded that discontinuous drinking of a moderate amount of ethanol can be more harmful for the immune system than a continuous ethanol intake, as the model applied in the present study. Those authors suggested that probably the discontinuous alcohol intake induces a greater stress as indicated by the searched immune indicators.

Therefore, future studies must be carried as to elucidate the isolate and combined effects of alcohol and stress over periodontal tissues. Our data suggest that although stress and alcohol may be harmful separately, their association probably triggered different processes. The evaluation of these factors is relevant because the combination of stress and alcohol drinking is a frequent condition in human beings with periodontitis. Although the association did not result as expected, the effects of alcohol drinking confirmed to be an important factor for the development of a periodontal disease.

## 5. Conclusions

Stressed animals which drank alcohol showed worse periodontal status than negative controls. However, nonstressed animals who also consumed alcohol for the same period showed a pattern of periodontal breakdown even more severe. Therefore, the factors of alcohol drinking and stress do not seem to show any synergic effect. 

## Figures and Tables

**Table 1:** Mean values (mm) of loss of histological attachment (CEJ-JE) and bone loss (CEJ-BC) for the different groups.

	CEJ-JE	CJE-BC
	(mean ± SD)	(mean ± SD)
Stress + alcohol	0.33 ± 0.09^a^	0.61 ± 0.03^a^
Nonstress + alcohol	0.77 ± 0.27^b^	1.09 ± 0.32^b^
Positive control	0.25 ± 0.16^a^	0.92 ± 0.20^a^
Negative control	0.06 ± 0.14^c^	0.21 ± 0.02^c^

Different letters within the same column indicate statistically significant differences between the groups (*P* < 0.05). CEJ-JE: loss of histological attachment; CEJ-BC: bone loss.
